# Sexual dimorphism in response to repetitive bouts of acute aerobic exercise in rodents with type 1 diabetes mellitus

**DOI:** 10.1371/journal.pone.0273701

**Published:** 2022-09-09

**Authors:** Jordan C. Larocque, Silar Gardy, Mitchell Sammut, David P. McBey, C. W. James Melling

**Affiliations:** 1 School of Kinesiology, Western University, London, ON, Canada; 2 Department of Physiology and Pharmacology, Schulich School of Medicine, Western University, London, ON, Canada; Max Delbruck Centrum fur Molekulare Medizin Berlin Buch, GERMANY

## Abstract

The purpose of this study was to examine sex-specific differences in the blood glucose (BG) response to recurrent aerobic exercise in type 1 diabetes rats. Specifically, we examined the role of peak estrogen (E2) concentrations during proestrus on BG response to prolonged repetitive aerobic exercise. To do so, nineteen Sprague-Dawley rats were assigned to four exercised groups: control female (CXF; n = 5), control male (CXM; n = 5), diabetic female (DXF, n = 5) and diabetic male (DXM, n = 4). Diabetes was induced in DX groups via subcutaneous multiple injections of low dose streptozotocin (20mg/day for 7 days). After four days of exercise, muscle and liver glycogen content, liver gluconeogenic enzyme content, muscle Beta oxidation activity and BG responses to exercise were compared. The final bout of exercise took place during proestrus when E2 concentrations were at their highest in the female rats. During days 1–3 DXM had significantly lower BG concentrations during exercise than DXF. While both T1DM and non-T1DM females demonstrated higher hepatic G6Pase expression and muscle beta oxidation activity levels on day 4 exercise, no differences in BG response between the male and female T1DM rats were evident. Further, no differences in liver and muscle glycogen content following day 4 of exercise were seen between the sexes. These results would suggest that heightened E2 levels during proestrus may not be an important factor governing glucose counter regulatory response to exercise in female T1DM rats. Rather, the pre-exercise blood glucose levels are likely to be a large determinant of the blood glucose response to exercise in both male and female rats.

## Introduction

Type 1 diabetes mellitus (T1DM) is a disorder resulting from the autoimmune mediated destruction of the beta-cells in the pancreas. These cells are insulin producing, and their destruction results in sub-optimal levels of circulating endogenous insulin and elevated levels of blood glucose (hyperglycemia). Several health complications are associated with chronic hyperglycemia including retinopathy, nephropathy, and cardiovascular disease [1–3]. A large cohort trial, The Diabetes Control and Complications Trial (DCCT), demonstrated the importance of intensive insulin treatment therapy in the reduction of many of these diabetes-related complications, in comparison to the more traditional conventional insulin therapy treatment. However, intensive insulin therapy increases the risk for hypoglycemia onset three-fold in these patients [4–6], which can be further exacerbated with regular exercise [7]. The fear of exercise-related hypoglycemia is one of the most common barriers to attaining the recommended physical activity guidelines among the T1DM population [8].

To counteract rapid or gradual reductions in blood glucose during exercise, the body has regulatory systems to prevent or correct alterations in glucose homeostasis. This so-called glucose counter-regulatory (GCR) response is first characterized by the dissipation of circulating insulin followed by the secretion of several GCR hormones which ultimately act on hepatic tissues to stimulate glucose release [9]. Studies have indicated that each GCR hormone has a specific glycemic threshold at which they are released, with their recruitment occurring in a progressive fashion in the order of adrenal-epinephrine, glucagon, growth hormone and cortisol [10]. In patients without diabetes, antecedent bouts of hypoglycemia, induced by either exercise or pharmacologically administered insulin, have been shown to blunt or reduce the neuroendocrine and glucoregulatory responses to subsequent bouts of exercise [11, 12]. There is a sexual dimorphism in these hormonal responses to repetitive exercise, whereby the blunted hormonal response to subsequent exercise is more pronounced in males [11–13]. This blunted hormonal glucoregulatory response is evident in patients with T1DM and may exacerbate the risk of hypoglycemia development following repetitive exercise (see review [14]). It is unclear as to whether this risk of exercise-related hypoglycemia is sex-specific, as few studies have investigated the impact of antecedent or successive bouts of exercise in females with T1DM.

Circulating estrogen (E2) levels are highest during the luteal phase (LP) which is potentiated by exercise [15–17]. E2 plays a key role in maintaining glucose homeostasis in the body as higher levels of the hormone are linked with a lower respiratory exchange ratio (RER) and higher lipid oxidation [15, 18–20]. Further, the use of oral contraceptives in males and postmenopausal females resulting in elevated E2 levels has also been shown to increase circulating free fatty acid (FFA) during heavy aerobic exercise, suggesting an E2-mediated shift towards fat oxidation during the exercise bout [16, 17]. While E2 promotes lipolysis and increases fatty acid availability, the hormone works to decrease the rate of gluconeogenesis [21], sparing liver and muscle glycogen levels during exercise [19, 22]. It have been shown that E2 is a transcriptional repressor of phosphoenolpyruvate carboxylase kinase (PEPCK) and glucose 6-phosphatase (G6Pase) [23]. It is also documented that E2 may alter substrate utilization through systemic hormonal effects via the stimulation of exercise-mediated secretion of growth hormone (GH), a hormone which its primary effect in the body is to increase lipolysis and FFA mobilization [15, 16, 24]. Indeed, females have higher resting GH levels than males, and larger GH response to exercise is evident when E2 levels are highest [15, 17]. Due to the lower and relatively stable E2 levels during the follicular phase (FP) compared to LP and the potential metabolic influence of this hormone, studies are often conducted during the FP when E2 levels are the most stable and closer to (but still higher than) levels in males [11, 12, 24, 25]. In female patients with T1DM, these apparent fuel selection differences as a result of elevated E2 during the LP could have an impact on counterregulatory measures against rapid reductions in blood glucose. Moreover, this shift towards lipid oxidation could be glycogen sparing; ultimately, leading to the reduced risk of hypoglycemia in female patients with T1DM [15, 24].

The purpose of this study was to examine the sexual dimorphism in the blood glucose response to four recurrent bouts of prolonged aerobic exercise in T1DM and non-T1DM rats. The four-day moderate intensity (~75% VO_2_ max) exercise program was designed to progressively challenge the glycemic response to acute aerobic exercise through the alterations in both glucoregulatory hormonal responses and hepatic and muscle glycogen replenishment. Importantly, the exercise protocol was staged so that last day of exercise (day 4) whereby animals would be at greatest risk of hypoglycemia development coincided with the highest E2 levels during the estrous phase (LP in humans). To examine the glycogen sparing effect of E2 during recurrent exercise in female T1DM rats we examined hepatic and skeletal muscle fuel metabolism immediately after the fourth bout of exercise during the proestrus phase of the estrous cycle (day 4). We hypothesized that fuel selection differences during the high estrogen phase would result in a glycogen-sparing effect in the liver and skeletal muscle in both female T1DM and non-T1DM rats resulting in a minimal change in blood glucose levels during and following aerobic exercise. This glycogen sparing effect would not be evident in male counterparts leading to an increased hypoglycemic response in T1DM animals during the final day of exercise.

## Materials and methods

### Ethics approval

This study was approved by the University Council of Animal Care of Western University (London, Ontario, Canada) and conducted in accordance with the standards of the Canadian Council on Animal Care.

### Animals

Twenty Sprague Dawley rats (10 males, 10 females) were obtained from Charles River Laboratories (St. Constant, Que., Canada) at 8 weeks of age (56–62 days old). The rats were housed in standard cages in same sex pairs until assignment to groups at which point animals were housed in pairs with the fifth rat in each group housed singly. Rats were maintained on a light-dark cycle of 12 h (light 10am: dark 10pm), with temperature held at 20.5°C and relative humidity at 40%. Standard rat chow (% energy: protein  =  26%, carbohydrate  =  60%, fat  =  14%, enriched with vitamins and minerals; PROLAB RMH 3000, Brentwood, MO, USA) and water were provided ad libitum for the duration of the study. Throughout the experiment, animal handling was limited to three researchers and one animal care technician.

### Experimental groups

The numbers of animals requested in this study were arrived at using the Power Analysis function in SigmaStat (version2.03) and previously reported data examining the blood glucose response to exercise in T1DM rats [26]. Rats were randomly assigned and experimental treated within one of four groups: control (non-T1DM) exercise male (CXM, *n* = 5), control exercise female (CXF, *n* = 5), T1DM exercise male (DXM, *n* = 5), and T1DM exercise female (DXF, *n =* 5).

### Experimental design

#### Diabetes induction

**Fig 1:**
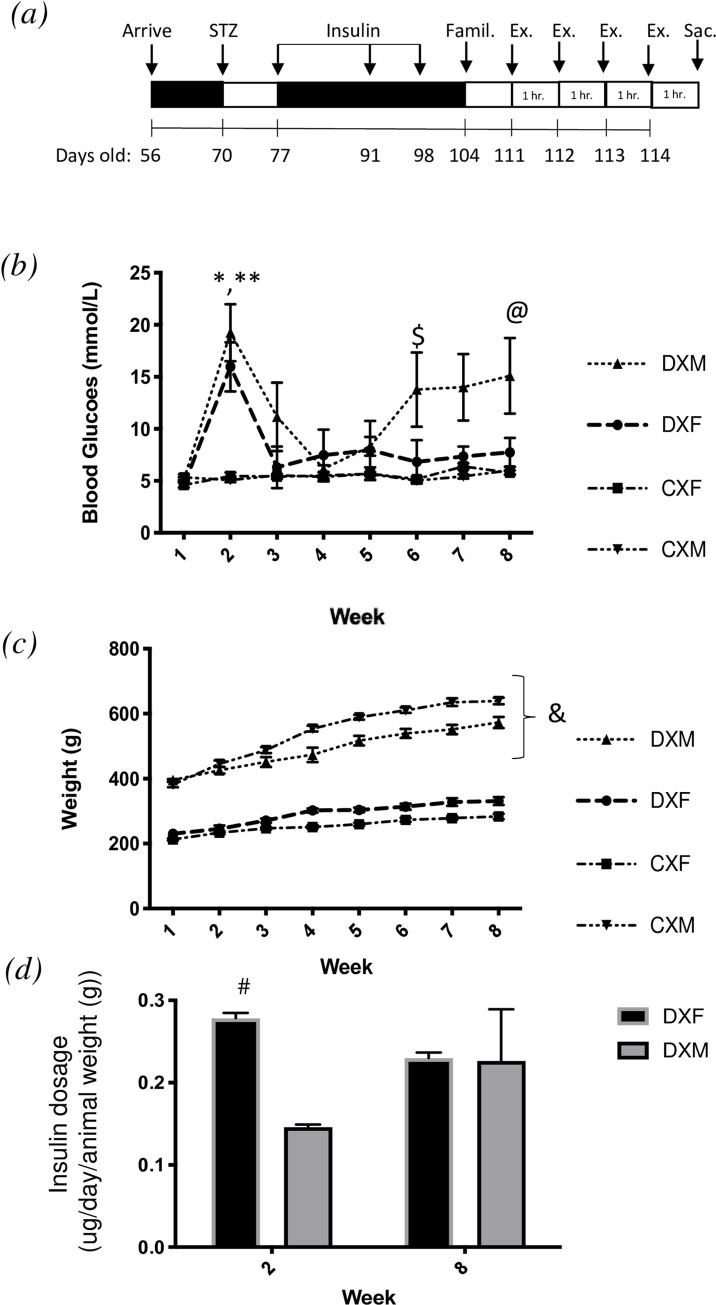
Weekly animal characteristics. (a) Experimental timeline. (b) *Mean non-fasted blood glucose (mM)*. *(c) Mean body mass (g)*. *(c) Insulin dose comparison between week 2 (diabetes induction;* 70–76 days old*) and week 8 (exercise protocol;* 111–117 days old*)*. *All data are presented as mean ± SEM*. * denotes DXM significantly different than CXM (P < 0.*05)*, ** denotes DXF significantly different than CXF and CXM (P < 0.*05)*, ^*$*^ *denotes DXM significantly different than CXM (P < 0*.*05)*, ^*@*^ *denotes DXM significantly different than CXF*, *(P < 0*.*05)*, ^*&*^ *denotes DXF and DXM significantly different than CXF and CXM*, *respectively (P < 0*.*05)*, ^*#*^ *denotes DXF significantly different than DXM at week two (*70–76 days old*; P < 0*.*05)*. *STZ*, *Streptozotocin; Famil*., *Familiarization; Ex*., *Exercise; Sac*., *Sacrifice; hr*., *hour*.

Animals were acclimatized for five days following transportation to the animal facility. Following this period, T1DM groups (DXF and DXM) underwent a 5-day protocol of intraperitoneal low dose injections of streptozotocin (STZ; Sigma-Aldrich) to induce T1DM (Experimental week two (70–76 days old), Fig 1B and 1C). For seven consecutive days, 20mg/kg of STZ (dissolved in a citrate buffer; 0.1M, pH 4.5) was injected each day and was administered within fifteen minutes of drug preparation. Diabetes was confirmed by two consecutive days of non-fasted blood glucose concentrations greater than 18mmol/L. Once diabetes was confirmed, insulin pellets were implanted (Experimental week three (77–83 days old), Fig 1B snd 1C*)* subcutaneously via surgical incision in the abdomen of all rats (1 pellet = 2 U insulin/per day). All animals in the diabetic groups were given one insulin pellet regardless of weight. Insulin therapy was then adjusted throughout the study by the addition or removal of pellets to maintain blood glucose within the range of 4–8 mmol/L to mirror standard treatment of intensive insulin therapy [27].

#### Exercise protocol

The recommended guidelines for forced treadmill running of rats were followed as reported by Garrigos et al. (2021) [28]. To minimize the stress generated by treadmill running, one week prior to exercise (Experimental week 7 (104–110 days old); Fig 1B and 1C) the animals undertook two familiarization sessions of 10 min. The familiarization of the males consisted of progressive running speeds (6% grade) at 15 m/min for 2 min, 18 m/min for 4 min, 20 m/min for 2 min, and 15 m/min for the last 2 min, while female sessions (6% grade) consisted of 15 m/min for 2 min, 24 m/min for 4 min, 27 m/min for 2 min, and 15 m/min for the last 2 min. All familiarization sessions were separated by 48 h and were performed 48 h prior to the acute bout of exercise. During the week of exercise (Experimental week 8 (111–117 days old); Fig 1B and 1C), all animals participated in 1 hour of aerobic exercise at 9am (end of dark cycle) for four consecutive days at approximately 75% of their maximal oxygen consumption. This time point of exercise each day was chosen to allow for the maximal levels of glycogen storage in tissues prior to exercise due to feeding throughout the day (dark cycle: 10pm-10am). To account for differences in maximal oxygen uptake and weight, the running speed for female groups was 25m/min at 6% grade and 18m/min at 6% grade for males. Treadmill speeds were based on studies reporting oxygen consumption values in exercising male and female rats [29]. Animals were removed from the exercise session (and excluded from experiment) if their running pattern become hopping in nature and their tails were not lifted while running on the treadmill. Animals were monitored during a recovery period of 60 minutes post-exercise (120 minutes from the initiation of exercise). Additionally, DXF and CXF were staged to ensure that the final day of exercise would be during the proestrus, high estrogen phase of the estrous cycle.

### Experimental measures

#### Blood glucose and body weight

Blood glucose levels and body weights were recorded weekly throughout the study (Fig 1B and 1C). Blood samples (~50 μL) were obtained via saphenous vein puncture in the hind leg. Blood glucose was measured using the Freestyle Lite Blood Glucose Monitoring System (Abbot Diabetes Care, INC.). During the week of exercise (Experimental week 8 (104–110 days old)), blood samples were collected on day 1, prior to commencing exercise, at 30 min intervals until 60 min post-exercise. On day 4, blood samples were collected prior to commencing exercise (0 min), 30 min, and 60 min of exercise. Blood was collected in non-coated tubes then centrifuged at 3000rpm for 20 min at 4°C. Supernatant was obtained and stored at -80°C until analysis.

#### Vaginal swabs

**Fig 2:**
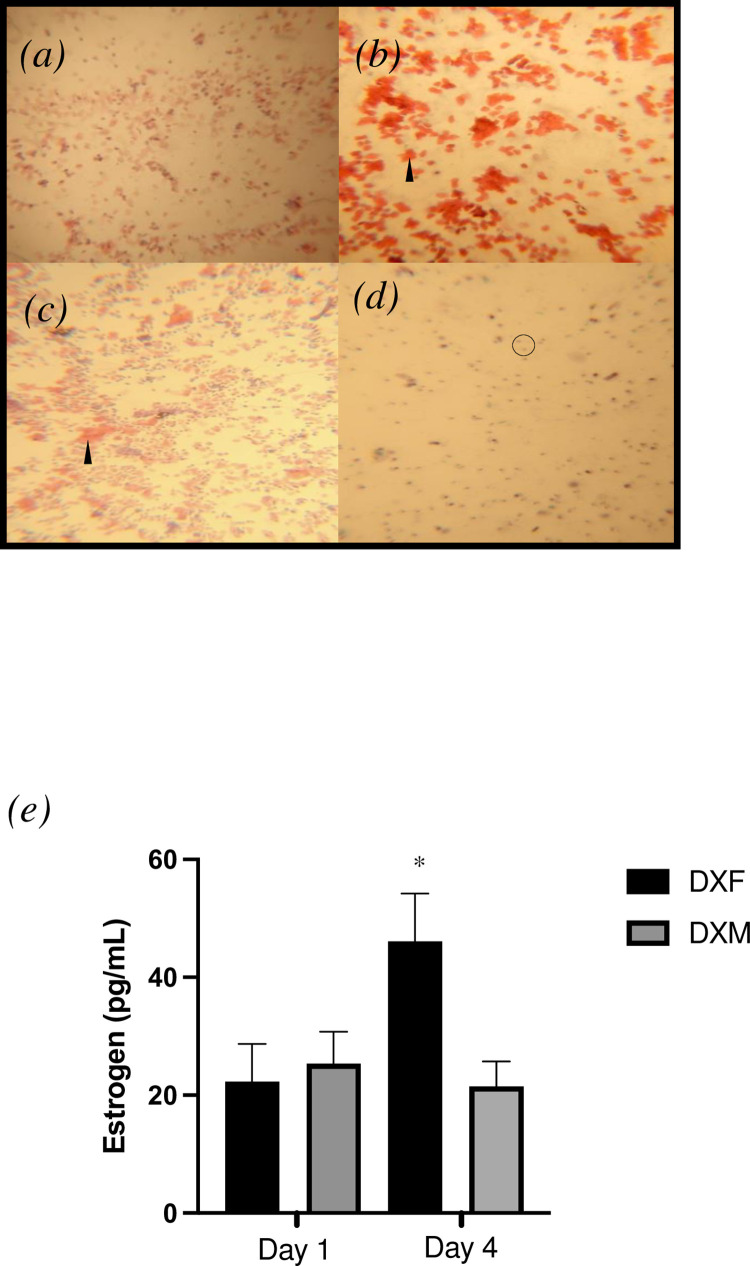
Estrous cycle determination. Vaginal smears of (a) proestrus, (b) estrous, (c)metestrus and (d) diestrus. Nucleated epithelial cells (white triangle) and clumping are present in proestrus. Keratinized epithelial cells (black triangles) are predominant in estrous. Neutrophils (circled) are highly present in diestrus. (e) Serum estrogen (pg/mL) on day one and four. All data are presented as mean ± SEM. * denotes day 4 DXF significantly different than DXF (day 1), DXM (day 1) and DXM (day 4) (P < 0.05).

In Week 2 (70–76 days old) and 3 (77–83 days old) of the experiment (Fig 1B and 1C), DXF and CXF underwent daily vaginal swabs for twelve days to determine the length of the estrous cycle (Fig 2A–2D). A sterile cotton swab was dipped in saline (0.9 M) and inserted into the vagina at a 45° angle, half turned, then removed. The swabs were then rolled horizontally onto a clean slide with firm pressure. Animals underwent additional swabbing once per week to confirm estrous stage prediction, including the week prior to exercise. Upon determination of the length of the estrous cycle, females were staged such that the last day of exercise occurred during proestrus.

#### Tissue collection

Rats were euthanized within five minutes of the final bout of exercise to allow for liver and skeletal muscle tissues to be examined (day 4). Animals were placed under 5% isoflurane until all reflexes were absent and euthanized via cardiac puncture. Liver and skeletal muscle tissue (red vastus muscle) was obtained and frozen in liquid nitrogen, then stored at -80°C until analysis.

#### Hematoxylin and eosin staining

Vaginal slides were dried for at least five minutes, then placed in a fixing solution (Rapid Fixx, Shandol) for one minute. Slides were placed in Harris Haematoxylin for 1 minute, then rinsed in tap water for 1 minute. Excess stain was removed with minimal amounts of water and dehydrated in 70% ethanol for 1 minute. Slides were submerged in 5% Eosin solution for 1 minute. Slides were rinsed again in tap water to remove excess stain and dehydrated in ascending alcohol. Finally, slides were cleared using xylenes and mounted using toluene-based media. Following staining slides were examined under a Zeiss AxioVert S100 microscope at 10x magnification to determine the phase of estrous (Fig 2A–2D).

#### Estrogen and growth hormone quantification

**Fig 3:**
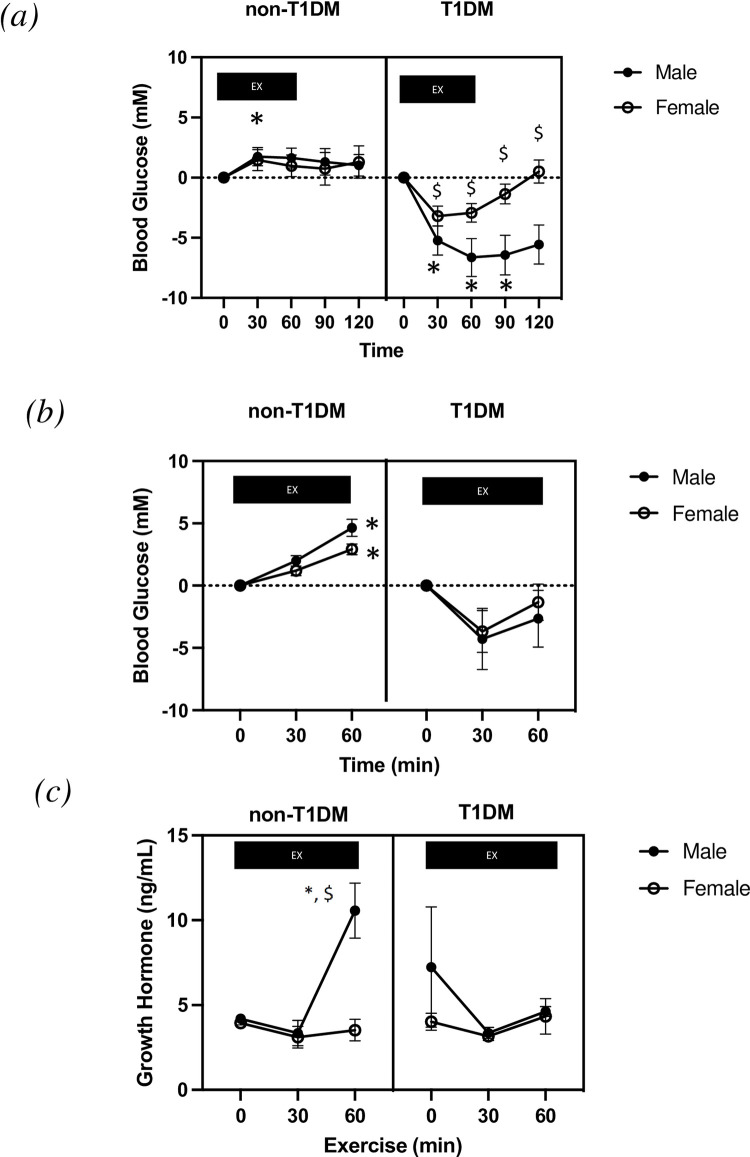
Blood glucose responses to exercise. Delta blood glucose data (mmol/L) from (ac) days 1–3 and (bd) day 4 as well as (c) day 4 serum growth hormone levels. All data are presented as mean ± SEM. * denotes significantly different from baseline values (0min; P < 0.05). $ denotes significantly different between DXF and time matched DXM. @ denotes significantly different than CXM, CXF, and DXF (P < 0.05).

Serum estrogen (Fig 2E) and growth hormone concentrations (Fig 3C) were determined via an enzyme-linked immunosorbent assays (17-beta Estradiol and Growth Hormone Rat ELISA Kits, Abcam) as per the manufacturer’s instructions. Estrogen levels were measured on resting samples obtained from the day 1 and day 4 via a saphenous vein blood draw, while growth hormone levels were measured on blood samples obtained at 0 min, 30 min and 60 min of exercise on day 4.

#### G6Pase and PEPCK expression

Liver samples were removed from storage at -80°C and kept on ice. Liver tissues were weighed (20 mg) and submerged in lysis buffer (15 mM Tris pH = 7.0, 600 mM NaCl and 0.1mM EDTA) for a 1:10 weight-volume (w/v) ratio of tissue to buffer. The buffer-tissue mixture was kept on ice while being subjected to three, 1–3 second pulses by a basic mechanical homogenizer (IKA Laboratories). Homogenized samples were transferred into 1.5 ml Eppendorf tubes and put on a shaker at 4°C for 2 hours. Afterwards, samples were centrifuged at 4°C at 12000 rpm for 20 min, followed by extraction of supernatant. A Bradford protein assay (Bio-Rad) was used to determine protein concentration. Protein samples were prepared with an equal volume of 2x Laemmli SDS-PAGE (4% SDS, 20% Glycerol, 10% β-mercaptoethanol, 0.015% bromophenol blue, 0.125M Tris, pH 6.8) and submerged in a 90°C water bath for 5 min. 15 ug protein was loaded into 12% polyacrylamide gels and ran at 140V for 2 hours. Gels were then transferred on nitrocellulose membranes (Bio-Rad) at 4°C at 70V for 1.5 hours. Following transfer, membranes were washed in TBS-T (Tris Buffer Saline, 0.1% Tween-20) for 5 minutes, 3 times. Membranes were then blocked with a 5% w/v solution of TBS-T and skim milk powder for 1 hour before being washed in TBS-T, 3 times, 5 minutes each time. Once washed, membranes were incubated for 2 hours at room temperature with primary antibodies detecting: anti-B-actin (ab8227; Abcam, Cambridge, UK), anti-PEPCK (ab70358; Abcam, Cambridge, UK), and anti-G-6-pase (ab93857; Abcam, Cambridge, UK) per manufacturer’s instructions. The following day, primary antibody was removed, and membranes were washed for 10 minutes in TBS-T, 3 times before being incubated with a 5% w/v solution of skim milk powder, TBS-T and secondary antibody (#170–6515 Goat pAB anti-rabbit IgG HRP conjugate; BioRad, Hercules, CA, USA) at a 1:20000 dilution for 2 hours at room temperature. After incubation, membranes were washed in TBS-T for 10 minutes, 3 times. Washed membranes were then prepared in Bio-Rad chemiluminescence substrate and images were captured using the BioRad Chemidoc MP System.

#### Glycogen content and B-oxidation activity

For the glycogen assay, approximately 20 mg of muscle or liver tissue was placed into a 2.0 mL eppendorf tube and samples were submerged in 30% potassium hydroxide solution saturated with sodium sulfate, and placed in boiling water for 30 min. Samples were precipitated in 95% ethanol for 30 min in an ice bath, followed by centrifugation at 3000 rpm for 30 min. The supernatant was discarded, and the glycogen pellet immediately dissolved in ddH_2_O. Homogenized tissue samples were added to a 96 well uncoated plate. A colour reaction was developed by rapid addition of 5% phenol and 98% sulfuric acid to the sample. Samples were placed in a water bath (37°C) for 20 min. Samples were analyzed in triplicate at a 490 nm on a microplate reader and compared to known glycogen standards.

For the Beta-oxidation assay, muscle (red vastus lateralis) samples were removed from storage at -80°C and kept on ice. Approximately 30 mg of tissue was placed into a 1.5 mL eppendorf tube. Samples were submerged in buffer (5 mM potassium dihydrogen orthophosphate (K_2_HPO_4_), 1 mM EDTA, 0.1 mM DTT; pH of 7.4) for a 1:10 weight-volume (w/v) ratio of tissue to buffer. The buffer-muscle tissue mixture was kept on ice while being subjected to three, 1–3 second pulses by a basic mechanical homogenizer (IKA Laboratories). Homogenized muscle samples were added to a quartz cuvette with assay buffer (1M Tris-HCl, pH 7.0, 0.5 M EDTA, pH 8.0, 10% Triton X-100) and 5mM NADH. The mixture was incubated for 4 minutes at 30°C allowing for mitochondrial permeability. Following an additional 1 minute after incubation the reaction was initiated by adding 5mM acetoacetyl CoA. The sample cuvette was vortexed, and the reaction was read for 2 minutes in 30 second intervals on a NanoDrop2000 C Spectrophotometer (Waltham, MA, USA) at 340 nm.

### Data analysis

Weekly blood glucose levels and body weights were compared using a three-way analysis of variance (ANOVA) with sex, time and diabetes as factors. Estradiol concentration on day 1 (diestrus) and day 4 (proestrus), as well as western blotting data (G6Pase, PEPCK), glycogen concentration and beta-oxidation levels were analyzed using a two-way ANOVA. Delta blood glucose and growth hormone levels during (and following) exercise were compared using a repeated measures ANOVA with sex (and time) as the factor. Post-hoc analysis was performed using Tukey’s multiple comparisons test when significant differences were found. Statistical analysis was completed using GraphPad Prism 8 (GraphPad Software, Inc.). Significance was accepted at an alpha value of 0.05.

## Results

### Animal characteristics

Results of both weekly blood glucose (Fig 1A) and body weight (Fig 1B) were analyzed to examine the influence of diabetes (T1DM vs. non-T1DM), time (week of study), and sex (male vs. female). One animal from the DXM was excluded as they were unable to complete the 4-day exercise protocol (see exclusion criteria for treadmill running in Materials and Methods). There was a significant interaction between time and diabetes (P < 0.0001), and time, diabetes and sex (P < 0.05). In week two (70–76 days old) of the study, DXF and DXM had higher blood glucose levels than CXM and CXF (P < 0.0001). There was no difference in week two (70–76 days old) blood glucose levels between DXF and DXM (P > 0.05). At week six (98–104 days old), blood glucose levels were significantly elevated in DXM in comparison to CXM (P < 0.05). At week eight (111–117 days old), blood glucose was significantly higher in DXM compared to both CXM and CXF (P < 0.05).

The effects of sex and week on body mass were statistically significant (P < 0.0001) while diabetes was not (P > 0.05). There was an interaction between time and diabetes (P < 0.01), time and sex (P < 0.0001), diabetes and sex (P < 0.0001), and time, diabetes and sex (P < 0.0001). The body mass of DXM and CXM were significantly higher than that of DXF and CXF, respectively (P < 0.0001). No differences in body mass between DXM and CXM (P > 0.05) and DXF and CXF (P > 0.05) was evident.

The initial dosage (week 2; (70–77 days old)) of insulin (ug/day/animal weight) was higher in DXF (P < 0.05) primarily due to the smaller size of the females (Fig 1C). There was no difference in insulin dosage between the groups in week 8 (111–117 days old) of the study (P > 0.05).

### Vaginal swabs

Representative vaginal images of each estrous stage are shown in Fig 2A–2D. Proestrus (Fig 2A) shows nucleated cells that present in clumps or sheets (arrow). Estrous (Fig 2B) shows an abundance of keratinized, denucleated epithelial cells (arrow). Metestrus (Fig 2C) and diestrus (Fig 2D) both show some nucleated cells and neutrophils, with metestrus showing higher cellularity (circled) than diestrus. Slides were stained using a standard hematoxylin and eosin protocol and examined against known examples of the stages of estrous for neutrophil content, the presence and number of keratinized cells, and the presence of mucus among other indicators.

Estrogen concentrations measured using ELISA are shown in Fig 2E. There was a significant effect of time and an interaction between time and sex (P < 0.05). Blood serum was obtained prior to day 1 and day 4 of exercise (t = 0min). As confirmed by vaginal cytology, day 1 serum was obtained during estrous, and day 4 serum was obtained during proestrus. Day 4 DXF had significantly higher estrogen than both day 1 DXF and day 4 DXM. There was no difference in estrogen concentration on day 1 between DXF and DXM (P > 0.5). Day 1 and day 4 estrogen concentration was not different in DXM (P > 0.5).

### Blood glucose response to exercise

The delta blood glucose responses to exercise are presented in Fig 3A and 3B. Results from the first three days (estrous, metestrus and diestrus) of exercise are presented together in Fig 3A as animals demonstrated similar blood glucose response to each exercise bout during these days (*S1 Fig*). There was a significant effect of time (P < 0.05) and a significant interaction between time and diabetes (P < 0.0001). Pre-exercise blood glucose (0min; BG) levels in CXM and CXF were significantly lower in comparison to 30 min of exercise (P < 0.05), but BG returned to normal resting BG by 60 min. At 30 min of exercise DXM blood glucose levels were significantly lower than pre-exercise levels which remained significantly lower in the recovery from exercise including 120 min post exercise. DXF blood glucose were significantly higher that DXM at 30min, 60 min, 90 min and at 120 min (60 minutes post exercise). In contrast to DXM, BG values in DXF were not significantly different at any time point in comparison to pre-exercise values.

Day 4 BG levels were obtained at pre-exercise (0 min) and 30 min and 60 min of exercise (Fig 3B). Both CXM and CXF demonstrated a significant increase in blood glucose levels in response to exercise at 60 min (versus pre-exercise levels) which was not different between sexes. There were no differences in blood glucose between DXM and DXF at any time point (P > 0.05). There was a main effect of time (P < 0.05) and an interaction between time and diabetes (P < 0.05).

**Fig 4:**
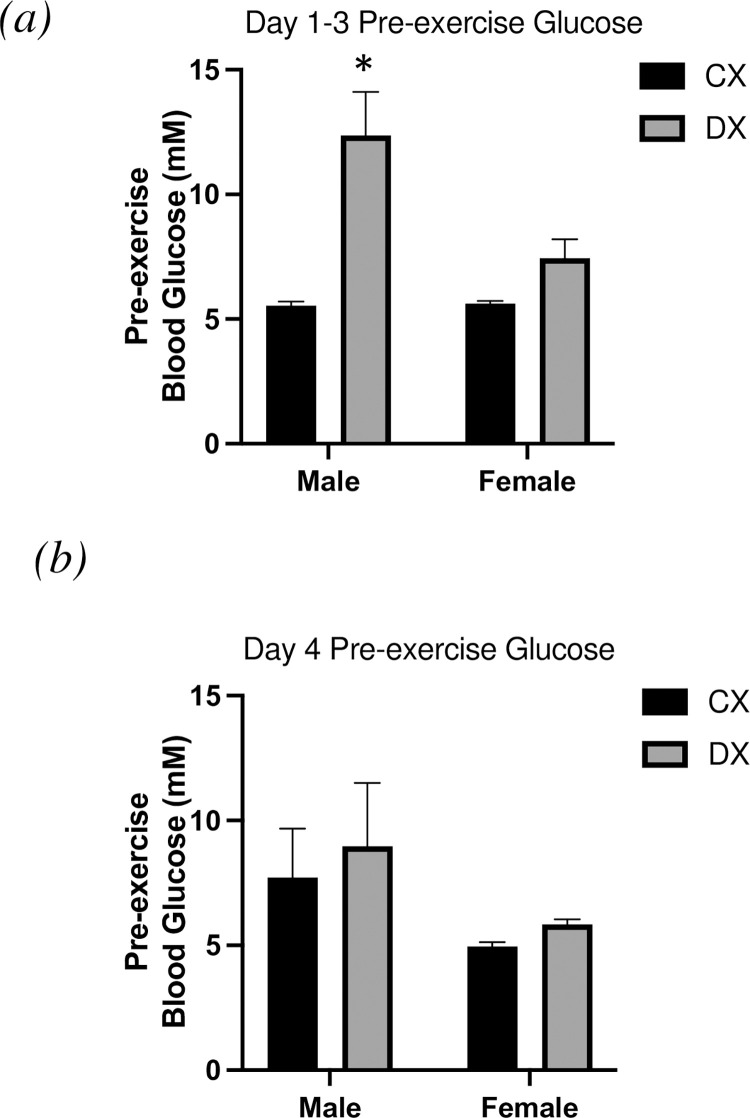
Pre-exercise blood glucose. Pre-exercise blood glucose data (mM) from (a) days 1–3 and (b) day. All data are presented as mean ± SEM. * denotes significantly different from CXM, CXF, and DXF (P < 0.05).

Absolute pre-exercise (0 min) blood glucose levels are presented in Fig 4A and 4B. On day 1–3 there was a main effect of sex (P < 0.05) and diabetes (P <0.05) on blood glucose levels. There was an interaction between sex and diabetes (P > 0.05). DXM had significantly higher pre-exercise blood glucose levels in comparison to DXF, CXM and CXF.

### Growth hormone concentration

Serum growth hormone levels were obtained at pre-exercise (0 min) and 30 min and 60 min of exercise (Fig 3E) on day 4. After 60 minutes of exercise, CXF were significantly higher (P < 0.05) than their male counterparts (CXM). There were no differences in growth hormone levels at any time point in the DXM and DXF groups. There was a main effect of time (P < 0.05) and an interaction between time and diabetes (P < 0.0001).

### Glycogen content and Beta-oxidation activity

**Fig 5:**
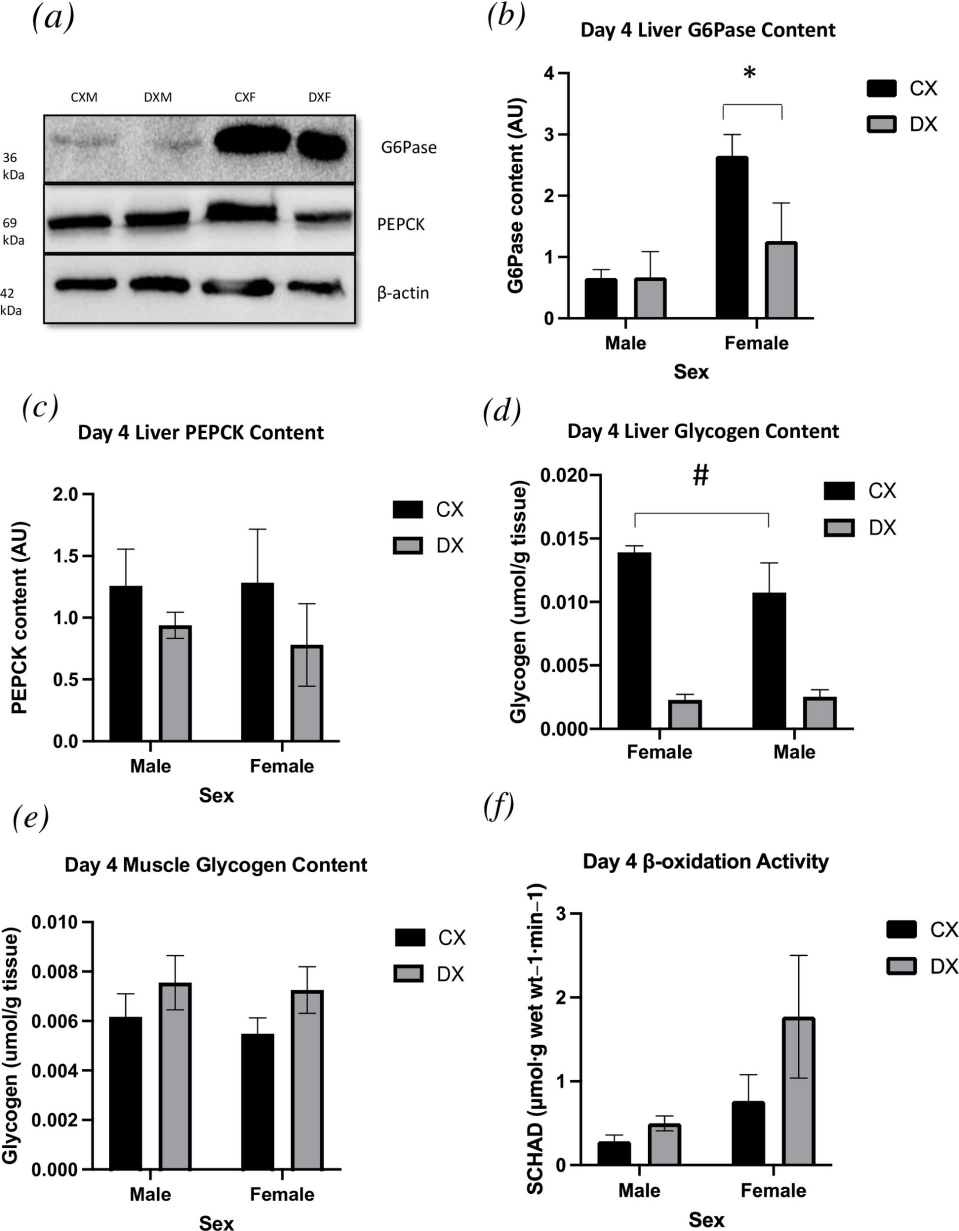
Western blotting data. (a) Representative blots and quantification of liver gluconeogenic enzymes (b) G6Pase and (c) PEPCK and (d) liver and (e) muscle, as well as SCHAD (f; beta-oxidation) activity in skeletal muscle on day four following the final bout of exercise. All data are presented as mean ± SEM. * denotes significant main effect for sex (P < 0.05). # denotes significant main effect for diabetes (P < 0.05).

Liver and muscle samples were taken immediately following the final bout of exercise on the fourth day. In liver tissue, there was a main effect of diabetes (P < 0.05) and no interaction between sex and diabetes (P > 0.05) (Fig 5D). Liver glycogen was significantly lower in DXF compared to CXF (P < 0.05). DXM also had significantly lower liver glycogen content than CXM (P < 0.05). Muscle glycogen content was assessed in skeletal muscle (red vastus lateralis) and demonstrated no effect of sex or diabetes (Fig 5E; P > 0.05). There was no interaction between sex and diabetes (P > 0.05).

Beta-oxidation, short-chain β-hydroxyacyl-CoA dehydrogenase (SCHAD) activity was assessed in lysates of skeletal muscle (vastus lateralis) using the SCHAD assay (Fig 5F). There was a main effect of sex (P < 0.05) and no interaction between sex and diabetes (P > 0.05).

### Western blotting analysis

Liver samples were homogenized for protein collection and subsequent western blot analysis for the detection of G6Pase and PEPCK was completed (Fig 5A–5C). Protein quantification was measured in arbitrary units (AU) using ImageJ software. There was a main effect of sex (P < 0.05) for G6Pase but no interaction between sex and diabetes (P > 0.05). No significant differences were observed in PEPCK content between groups (p>0.05).

## Discussion

Much of the research examining the benefits of regular exercise in T1DM continues to focus on male subjects, and studies that do utilize female subjects, typically use physiological states of low hormonal levels (follicular phase) or experiments whereby hormones are experimentally regulated [11, 12, 16, 17, 25]. Estrogen (E2) is known to impact the progression and risk factors associated with many other diseases, yet few studies have investigated the effect of E2 on the metabolic complications associated with T1DM. Indeed, E2 levels are correlated with a shift in fuel usage towards higher fat oxidation, and therefore elevated levels of this hormone may lessen the risk of hypoglycemia development in female patients with T1DM during exercise. Moreover, experimental studies need examine the metabolic effects of E2 on glucose metabolism during the correct stages of adulthood where E2 levels are elevated. It has been shown that serum estradiol levels in female rats reach adulthood levels at the postnatal age of 48 days [30]. Importantly, it has been shown that exercise-related changes in body composition of rats occur when estradiol levels begin to increase [31]. As such, this study aimed to examine whether the blood glucose response to repetitive exercise is different between adult (> 100 days old) male and female T1DM and non-T1DM rodents. Moreover, we examined if the higher levels of E2 during the proestrus phase of the estrous cycle would mitigate the reduction in blood glucose during exercise in T1DM female rats.

The primary finding of the current study is that male and female T1DM rats demonstrated a significantly different BG response to acute exercise on days 1–3 (low E2) but did not show differences on day 4. On day 1–3 of exercise, DXM animals demonstrated a significant reduction in BG at 30 min of aerobic exercise that was maintained until 120 min (60 min post exercise). DXF on the other hand, demonstrated significantly elevated levels on BG in comparison to their male T1DM counterparts (DXM) at each of these time points. Further, the BG levels in DXF were not different at any time point in comparison to pre-exercise levels (Fig 3A). The reduction in blood glucose levels during exercise in male T1DM rats is consistent with previous studies from our lab [26, 32]. However, this would be the first evidence that female T1DM rats do not exhibit the same magnitude drop in BG during exercise in comparison to their male counterparts.

Contrary to our hypothesis, we anticipated that the reduction in BG levels in female T1DM rats with exercise would be evident during the final bout of exercise (day 4), due to increased levels of E2 in the proestrus stage of estrous. However, the sex differences were evident during the earlier bouts of exercise when estrogen levels were low and comparable to males. The blood glucose response to exercise on day 4 was similar between male and female T1DM rats independent of the phase of the estrous cycle. In fact, it was the male T1DM rats that demonstrated an altered blood glucose response to exercise on day 4, suggesting that factors other than heightened E2 are responsible for the sex-mediated difference to glucose control during exercise. Riddell et al. reported that higher pre-exercise glucose levels in adolescents exhibit a greater absolute drop in blood glucose levels during exercise than those individuals that exercised at an euglycemic range [33]. Patients with T1DM often consciously elevate pre-exercise glucose levels to combat the risk of hypoglycemia by excessively decreasing insulin and subsequently compromising glycemia through carbohydrate intake [34, 35]. In the current study male T1DM animals had a significantly higher pre-exercise blood glucose levels on day 1–3, despite a similar insulin dosage to that of female T1DM animals. It has been shown that in the hyperglycemia state fuel metabolism in patients with T1DM is dominated by carbohydrate oxidization [36]. Even in the presence of identical insulin concentrations hyperglycemia is shown to increase intramuscular glycogen usage. On the contrary, substrate oxidation in patients with T1DM performing aerobic exercise in euglycemia is similar to non-diabetic individuals demonstrating a shift towards lipid oxidation and intramuscular glycogen sparing [36]. We demonstrate that on day 4 that both males and females T1DM animals exhibit near normal levels of blood glucose pre-exercise and a mirrored blood glucose response to aerobic exercise. These results would suggest that pre-exercise levels of blood glucose are a strong determinant of BG response to exercise independent of sex and hormonal levels.

It has been shown that an E2-mediated shift in fuel selection towards fat during aerobic exercise protects against liver glycogen depletion and therefore smaller post exercise glucose uptake [24, 37]. The post-exercise period following exercise of such an intensity is when patients with T1DM face the greatest risk of hypoglycemia as the body attempts to reestablish glucose homeostasis [32, 38]. We hypothesized that by performing the final bout of exercise during proestrus stage liver and/or muscle glycogen would be spared in both DXF and CXF compared to DXM and CXM due to an increase in E2-mediated fat oxidation. While both T1DM and non-T1DM females demonstrated significantly higher β-oxidation activity in skeletal muscle immediately after exercise compared to males; we found no differences between the sexes in glycogen content in these tissues (Fig 4). The only significant factor in liver glycogen content was diabetes, which was to be expected in males based on previous literature from our laboratory [26, 32, 39]. The absence of a glycogen sparing effect in females contrasts with data showing lower glycogen usage in healthy exercising women in the LP [15] due to the higher E2 levels. However, this menstrual phase effect on sparing muscle glycogen content were conducted in isolated bouts of exercise, therefore it can be postulated there was no muscle glycogen differences between the sexes was due to the exhaustive nature of the prescribed exercise protocol. Successive days of exercise have demonstrated to produce a marked reduction and utilization of muscle glycogen content [40]. Indeed, muscle glycogen content on day 4 post-exercise ranged from approximately 0.25–0.64 g/100g which is substantially lower than previously reported muscle glycogen content post-exercise [26, 39]. Moreover, post-exercise muscle glycogen stores can take greater than 24 hours for complete restoration to near pre-exercise levels [41]. It is plausible that the successive exercise protocol did not allow for full resynthesis of muscle glycogen stores and thus a sparing effect of E2 on these tissues was not achieve.

The liver is a direct target of E2 due to the high expression of E2 receptor alpha, which has been well characterized to impact lipid and glucose metabolism [42]. E2 has been shown to modulate fuel selection through the pituitary release of growth hormone (GH) secretion [42]. It has been shown that GH impedes the actions of insulin and promotes fat mobilization, FFA circulation and glycerol levels. Further, in skeletal muscle, GH increase TAG storage and lipid oxidation [43]. In the current study we see a sexual dimorphism in the secretion of GH in non-T1DM rats whereby females showed greater increase with exercise on day 4 (corresponding to high E2). This is consistent with previous reports that GH response is higher in females that men following higher levels during intense exercise [44, 45]. In contrast, exercise mediated GH secretion was not evident on day 4 of female T1DM rats which may suggest that T1DM impacts the E2 mediated activation of GH secretion. It is plausible that the previous bouts of exercise stressed the glucoregulatory response system of DXF rodents such that the glucose sparing effects of E2 were mitigated. It has been shown that prior aerobic exercise can blunt the GH response to subsequent exercise in T1DM patients [46]. Moreover, other glucose-regulatory hormonal factors may be involved in the sex-related differences in glucose regulation to exercise independent of E2. We have previously shown that T1DM male animals demonstrate a blunted (lower) glucagon response to exercise which is returned following 10 weeks of aerobic training [26]. It is not clear whether females demonstrate a normalized glucagon response to exercise; however, the lower insulin treatment levels in these female animals may reduce the negative impact of insulin on the release of glucagon during exercise.

The low levels of hepatic glycogen in females in comparison to males would suggest that glycogen was the preferred fuel source. Indeed, hepatic glycogen content is typically lower in populations with T1DM [47, 48]. Where the increases in BG levels during exercise in non-T1DM patients is almost entirely account for via glycogenolysis, patients with T1DM favour gluconeogenesis as the primary means for blood glucose replacement [49]. However, when hepatic glycogen stores become depleted (via 12 hr fast), gluconeogenesis is insufficient to match the high rates of peripheral glucose uptake during moderate and hard intensity exercise [50]. The decreased hepatic glycogen content in both male and femaleT1DM rats could have implications for combatting hypoglycemia, since hepatic glycogen is a prominent source of blood glucose during glucose-demanding states [51].

E2 and its receptor (ERalpha) have also been shown to stimulate lipid metabolism via the suppression of liver G6Pase and PEPCK gene activity in the fed state [52]. This in turn, forces the working muscle to rely heavily on fat stores due to the inability to generate new glucose via gluconeogenesis. It has been reported that diabetes-related hyperglycemia leads to an increase in gluconeogenic activity leading to elevated fasting glucose through and an increase in G6Pase and PEPCK a finding we have reported previously [26]. Interestingly, hepatic gluconeogenic rate limiting enzyme G6Pase was significantly higher in T1DM and non-T1DM females, but no difference in PEPCK content were evident between groups. Enhanced E2-induced lipid oxidation in females may suggests an increase in lipid availability leading and higher circulating gluconeogenic precursors glycerol and free fatty acids. The entry of glycerol into the gluconeogenic pathway bypasses the actions of PEPCK and thus only G6Pase content is primarily relevant for its conversion into glucose [53, 54]. Further work is needed using in vivo isotopic measurements assessing rates of gluconeogenesis and glycogenolysis to examine the sex specific role of GH (and E2), G6Pase expression and glycogen content following exercise and the impact of T1DM on this relationship.

In the final weeks of the study there was an increase in blood glucose in DXM relative to the other groups (Fig 1A), and this increased blood glucose was not responsive to additional exogenous insulin. While this increase in glucose in the males made it difficult to infer a direct relationship between E2 and glucose metabolism, this finding may highlight some sex differences in glucose control during long term intensive insulin therapy. Despite no direct measures of insulin resistance, it is plausible that the DXM cohort became resistant to insulin as evidenced by daily glucose levels outside of the intended target range (4–8 mM). Hyperinsulinemia may have elicited insulin resistance development through a mechanism of oxidative stress and may further impair the body’s insulin sensitivity response during exercise [55, 56]. The level of insulin in the DXM group was appropriate for the weight and size of the animals, and no such effect was seen in the female cohort who received the same amount of insulin relative to body size (Fig 1C). E2 may play a role in the sensitivity to insulin as sex differences in whole body insulin sensitivity are apparent [57, 58]. Moreover, it has also been shown that a decline in insulin sensitivity is paralleled to age-matched males following menopause [59]. Further work is needed to examine the sex specific discrepancies in insulin sensitivity between male and female T1DM animals and the potential role of E2. We and others have previously shown the importance of insulin sensitivity as a primary determinate of cardiovascular disease development in T1DM [60, 61].

A limitation of the current study is that a standardized treadmill protocol [29] based on age, weight, and sex of the animals was used to estimate exercise intensity (%VO_2_max) and was not tailored to the individual animal oxidative capacity as would occur in human exercise programs. Secondly, due to limited allowable blood sampling of the animals during and post exercise, blood insulin levels were not measured but were estimated based on pellets release rates and animal weights. Lastly, the time point of exercise at the end of the active cycle of the animal was chosen to allow for the maximal levels of glycogen storage in tissues prior to exercise due to feeding throughout the day. The metabolic response to exercise at this time point is unlikely to represent exercise at other (early) time points of the day. Indeed, it has been shown that prior early morning exercise leads to a different blood glucose response to exercise than afternoon exercise [62].

In conclusion, this study examined the effect of repetitive aerobic exercise on the glucoregulatory response to exercise in a male and female T1DM rat model. The current study found that there was a greater post-exercise blood glucose recovery in the DXF on days 1–3, suggesting that the DXF group was able to counteract changes in blood glucose in responses to exercise more successfully than DXM. However, the sexual dimorphism was not evident on the high E2 (day four of exercise) phase of the estrous cycle and there were no apparent differences in liver and skeletal muscle glycogen content between the male and female T1DM rats. This would suggest that E2 levels may not be an important factor governing glucose counter regulatory response to exercise in T1DM individuals. Rather, the pre-exercise blood glucose levels are likely to be a large determinant of the blood glucose response to exercise.

## Supporting information

S1 Raw images (PDF)

S1 Fig (PDF)
