# Supplementary figures and images for: Sexual dimorphism in response to repetitive bouts of acute aerobic exercise in rodents with type 1 diabetes mellitus
Source: PLoS One. 2022 Sep 9;17(9):e0273701. doi: 10.1371/journal.pone.0273701 (PMC9462568; doi:10.1371/journal.pone.0273701)

Supplemental data 1.

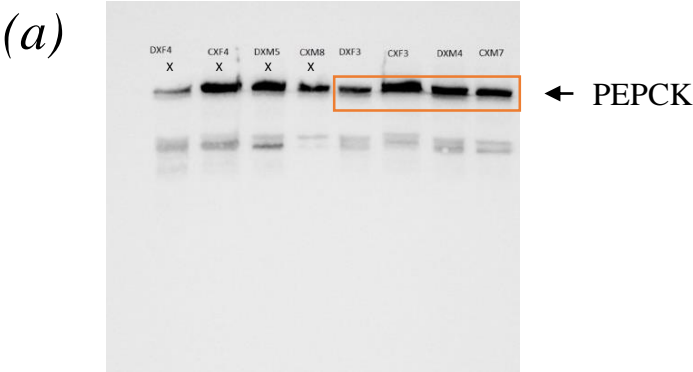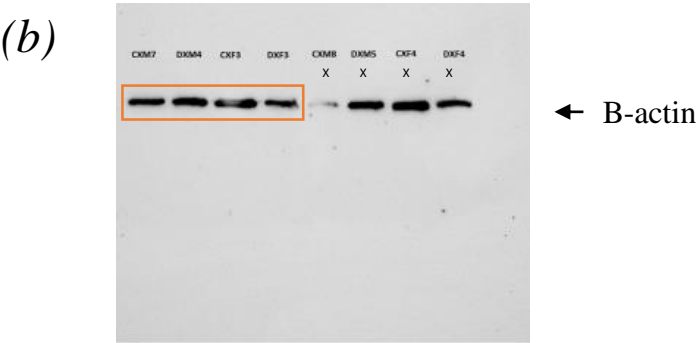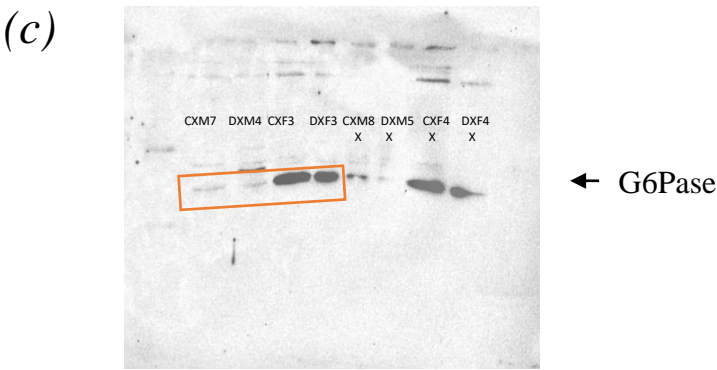

Supplement: S1 Raw images — (PDF) [file pone.0273701.s001.pdf]

Supplemental data 2.

(a)

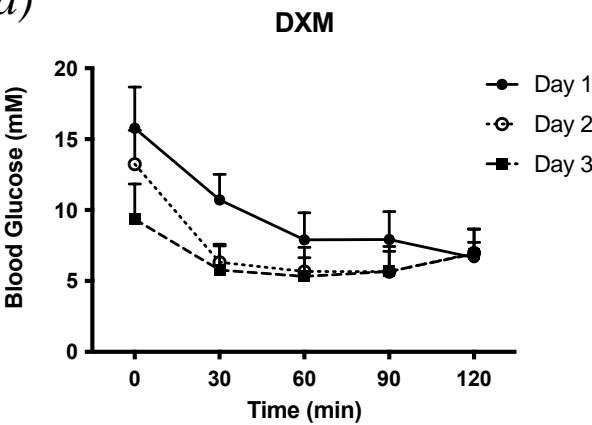

(b)

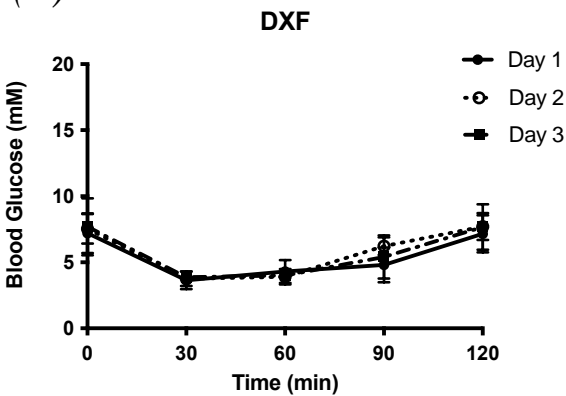

(c)

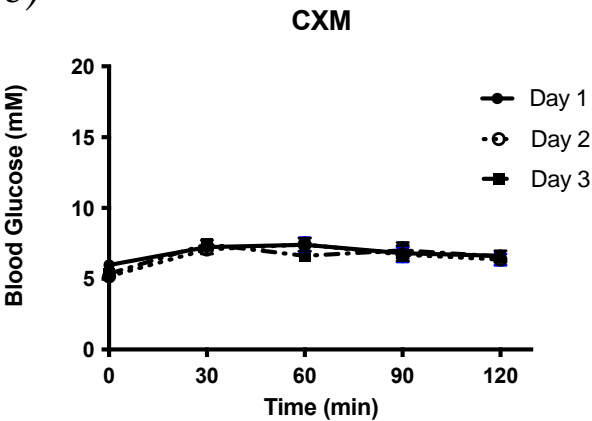

(d)

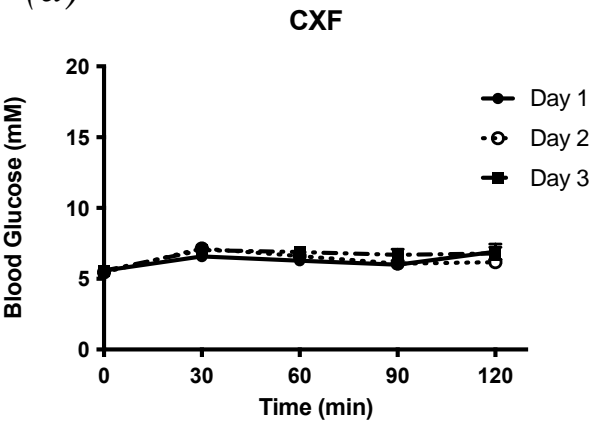

Supplement: S1 Fig — (PDF) [file pone.0273701.s002.pdf]
